# A modified stroke code for acute ischemic strokes during the coronavirus disease-2019 pandemic

**DOI:** 10.3325/cmj.2020.61.465

**Published:** 2020-10

**Authors:** Sohil Pothiawala

**Affiliations:** Department of Emergency Medicine, Woodlands Health Campus, Singapore

Coronavirus disease-2019 (COVID-19) was initially believed to manifest with predominant respiratory symptoms. However, emerging evidence points to neurological disease manifestations, with an increasing number of patients presenting with confirmed acute ischemic stroke (AIS) (1,2). The underlying pathophysiology of vascular thrombosis and subsequent AIS has been attributed to cytokine storm, a severe immune reaction leading to an increased inflammatory response and hypercoagulable state (3). AIS develops in approximately 5% of hospitalized COVID-19 patients, with a mortality rate of around 39% (4). Most ischemic strokes occur in older patients with traditional stroke risk factors and a 3-fold elevation in the initial D-dimer level (5). Here, the author would like to propose a modified emergency department (ED) stroke code for the evaluation of patients with confirmed or suspected COVID-19. A stroke code used in COVID-19 patients with a time-sensitive condition such as AIS needs to ensure not only rapid patient management, but also stroke team safety and the optimal and safe use of scan rooms and angio-suites.

## Rapid assessment in emergency department

**Figure 1:**
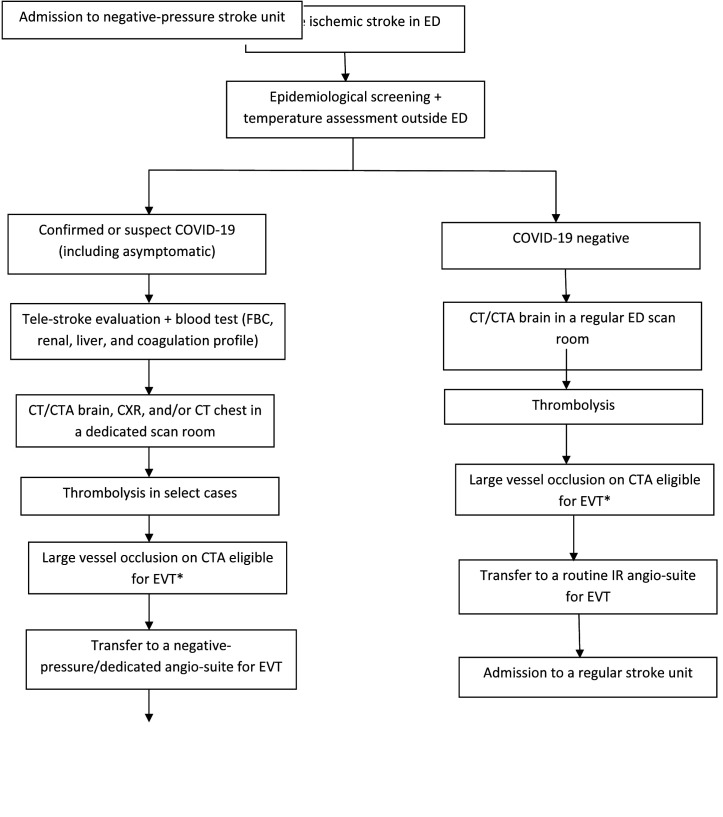
The proposed Modified Stroke Code during the coronavirus disease-2019 (COVID-19) pandemic. *Acute ischemic stroke (AIS) patients at primary stroke centers showing large vessel occlusion on computerized tomography angiography (CTA) eligible for endovascular thrombectomy (EVT) should be transferred to a comprehensive stroke center. ED – emergency department; FBC – full blood count; CXR – chest x-ray; IR – interventional radiology.

In the pre-pandemic times, many EDs have adopted an Acute Stroke Code, an algorithm facilitating a rapid assessment of patients presenting with signs and symptoms of AIS. The stroke code triggers the activation of the stroke team, which comprises of an ED specialist, neurologist, radiographers, and radiologist. During the COVID-19 pandemic, a need arose for a modified stroke code that would include COVID-19 screening (Figure 1).

The patients who fulfill any of the rapid COVID-19 screening criteria (positive travel history, positive history of contact with COVID-19 patients, acute respiratory infection symptoms, and increased body temperature) should be isolated already at the ED arrival, while the patients who develop AIS after a COVID-19 diagnosis should be isolated at the onset of stroke management. A challenge arises when an AIS patient presenting to the ED is asymptomatic, or when the neurological deficit is the primary presenting complaint of COVID-19. Another challenge is the patient's inability to provide an accurate travel or contact history at screening, or any history of stroke symptoms, due to altered mental state or aphasia. To overcome this problem, some centers recommend using laboratory tests, including full blood count, CRP, and D-dimer levels, along with reverse transcriptase polymerase chain reaction from nasopharyngeal and oropharyngeal swab test. However, the turn-around-time of these tests often exceeds the strict time frame for initial evaluation and management. Thus, all patients presenting to the ED with stroke may need to be evaluated as suspect COVID-19 patients.

To minimize the risk for health care workers during patient assessment and management, all personnel should wear adequate personal protective equipment (PPE) and maintain a distance of at least two meters from the patient, unless strictly necessary. Since stroke evaluation does not generate aerosols, the use of a positive air purified respirator may depend on its availability and institutional policy (1). The stroke team should comprise of a minimum number of health care providers and use telemedicine to review the evaluation process.

## Neuro-imaging in emergency department

AIS guidelines advocate non-contrast brain computerized tomography (CT) scan for every patient within 20 minutes of ED arrival (6). If the patient is confirmed to be COVID-19 negative, the imaging and further treatment can be carried out as per the usual hospital protocol. Confirmed or suspected COVID-19 patients should be sent to a dedicated imaging room. If a separate imaging facility is not available, imaging should be performed in a negative pressure carrier isolator, a machine made of non-porous vinyl material covering the patient that uses negative pressure and filters to prevent the contaminated air from escaping (1). After the isolator use, the entire scan room should be thoroughly disinfected.

Patients who are eligible for endovascular thrombectomy (EVT) need to undergo CT angiography (CTA) and perfusion imaging. Current guidelines recommend CTA before obtaining serum creatinine concentration in patients without a history of renal impairment (6). Patients with sepsis secondary to COVID-19 infection have a relatively high risk of renal insufficiency, and contrast exposure may lead to an increased risk of acute kidney injury and subsequent mortality. In patients who are not candidates for mechanical thrombectomy, the stroke team must analyze the risk-benefit ratio and avoid CTA and perfusion imaging. Concurrent pulmonary imaging using plain chest radiograph or a CT scan may aid in identifying radiological abnormalities suggestive of COVID-19. However, patients with an early stage of the infection may exhibit normal results, warranting a high level of suspicion.

## Management in the emergency department

### Intravenous thrombolysis

In patients with AIS, guidelines recommend the treatment with intravenous recombinant tissue plasminogen activator (rt-PA) up to 3-4.5 hours from symptom onset or from the time of the last known well (6). However, caution should be exercised in COVID-19 patients, who often present with fever, since fever can negatively affect clinical improvement after thrombolysis. In addition, many patients with COVID-19 sepsis often have leukocytosis with lymphopenia and elevated levels of C reactive protein (CRP) and D-dimer. Elevated leukocyte count and CRP on arrival and within 24 hours were associated with a poor three-month outcome after thrombolysis for AIS. Leukocytosis at baseline also increased the risk of symptomatic intracranial hemorrhage (ICH) post-thrombolysis (7). Elevated D-dimer level (median 1.4 μg/mL) was significantly associated with an unfavorable outcome and the occurrence of symptomatic ICH after thrombolysis (8). The extent of hepatic dysfunction secondary to COVID-19 infection is unknown, with liver damage and associated alteration of liver function tests (thrombocytopenia, elevated prothrombin time, international normalized ratio, and activated partial thromboplastin time) more likely to occur in patients with severe disease (9). Patients with hepatic dysfunction may have reduced intravenous rt-PA hepatic clearance, potentially resulting in increased serum levels and the risk of ICH (1).

While none of these laboratory results are an absolute contraindication to thrombolysis, in patients with ASI and suspected concomitant COVID-19 infection it is advisable to make a detailed assessment of coagulation profile before intravenous thrombolysis (1).

### Endovascular thrombectomy

Mechanical thrombectomy is recommended in AIS patients older than 18 years with an occlusion of the internal carotid artery or middle cerebral artery as identified on CTA, and with NIHSS score ≥6 and ASPECTS≥6. The procedure can be initiated within 6 hours of symptom onset. It is also recommended within 6-16 hours or 16-24 hours of the last known normal in a select group of AIS patients who meet additional eligibility criteria (6).

COVID-19 negative patients who need EVT can be immediately transferred to the hospital's interventional radiology (IR) angio-suite. However, suspect or positive COVID-19 patients need to undergo the procedure in a dedicated negative-pressure angio-suite. If a such suite is not available, there are guidelines on how to convert the existing angio-suites for COVID-19 patient management (10).

Every effort must be made so that EVT is performed without any delay, even during the pandemic. A modified stroke code that includes initial screening, serum creatinine measurement before contrast imaging to determine the risk of nephropathy, and an additional chest imaging using plain radiograph or chest CT may delay a rapid transfer of these patients from the ED to an angio-suite. A retrospective study evaluating the impact of a protected code stroke algorithm on EVT for acute stroke patients in the pandemic period showed that hospital arrival-to-puncture and-reperfusion time was 48.5 and 41 minutes longer respectively than in the pre-pandemic period. This delay is attributed to the additional screening procedure for COVID-19 (11). The long-term effect (using 90-day Modified Rankin Scale) of this delay on reperfusion needs further evaluation. Hence, given the multiple complexities associated with performing EVT in patients with suspected or confirmed COVID-19 infection, a strict protocol must be adopted to select AIS patients who would benefit most from mechanical thrombectomy on a case-by-case basis. AIS patients should then be admitted to a dedicated stroke unit with adequate equipment needed to monitor them in a negative-pressure room.

## Patient transfer to a dedicated stroke center

Not all hospitals are capable of managing patients with acute stroke, and there is an increasing trend of transferring these patients to a comprehensive stroke center for EVT and a higher level of care. Recommendations for the inter-hospital transfer of stroke patients vary, while the process may require special equipment and personnel, delaying EVT for 95-109 minutes (12,13). In the case of AIS patients with COVID-19 infection, the procedure is even more challenging, requiring additional resources and safety protection. Thus, the decision to transfer a patient to a comprehensive stroke center must be made on a case-by-case basis, and be limited to patients who would most likely benefit from it. In this scenario, the receiving center can make an appropriate patient selection by telestroke (6).

Even after the end of the current pandemic, instead of transferring patients requiring EVT to a comprehensive stroke center, more hospitals should be transformed into EVT capable centers, thus reducing the burden of transferring high-risk patients and achieving improved outcomes.

## Conclusion

Emergency physicians must have a high level of suspicion to identify AIS as an initial presentation of COVID-19. To address the challenges during this pandemic, a modified stroke code with a dedicated workflow needs to be established for screening, evaluation, and safe, coordinated management of AIS patients in the ED. The implementation of a strict screening process in the ED can help identify suspect or confirmed COVID-19 patients. The adherence to infection control measures by all members of the stroke team, a dedicated facility for neuro-imaging, and subsequent intervention in the angio-suite can ensure timely, safe, and optimal stroke management. This article proposes a modified stroke code addressing all these issues that can be individualized and adopted based on local institutional practice.
